# Metformin mitigates adipogenesis of fibro-adipogenic progenitors after rotator cuff tears via activating mTOR/ULK1-mediated autophagy

**DOI:** 10.1152/ajpcell.00034.2024

**Published:** 2024-04-08

**Authors:** Hao Zhou, Xingzuan Lin, Shujing Feng, Siyuan Zhu, Han Zhou, Huifang Chen, He Youwu, Zekai Wang, Ru Wang, Xiexiang Shao, Jianhua Wang

**Affiliations:** ^1^Xinhua Hospital Affiliated to Shanghai Jiao Tong University School of Medicine, Shanghai, People’s Republic of China; ^2^Department of Sports Medicine, School of Exercise and Health, Shanghai University of Sport, Shanghai, People’s Republic of China; ^3^Department of Hand Surgery, Huashan Hospital, Fudan University, Shanghai, People’s Republic of China; ^4^Department of Hand Plastic Surgery, The First People’s Hospital of Linping District, Hangzhou, People’s Republic of China; ^5^Department of Life Science, University of Toronto, Toronto, Ontario, Canada

**Keywords:** autophagy, fibro-adipogenic progenitors, mTOR/ULK1 signaling pathway, muscular fatty infiltration, rotator cuff tear

## Abstract

Muscular fatty infiltration is a common issue after rotator cuff tears (RCTs), which impair shoulder function. Females suffer a higher prevalence and a more severe degree of muscular fatty infiltration after RCT when compared with males, with the underlying mechanisms remaining unclear. Fibro-adipogenic progenitors (FAPs) are the primary source of muscular fatty infiltration following RCT. Our findings disclose that gender-specific disparities in muscular fatty infiltration are linked to mTOR/ULK1-mediated autophagy of FAPs. Decreased autophagic activity contributes to adipogenic differentiation in female FAPs after RCT. Furthermore, metformin could enhance mTOR/ULK1-mediated autophagic processes of FAPs, thereby alleviating fatty infiltration and improving shoulder functionality after RCT. Together, our study reveals that gender differences in muscular fatty infiltration arise from distinct autophagic activities. Metformin could be a promising noninvasive intervention to ameliorate muscular fatty infiltration of RCT.

**NEW & NOTEWORTHY** The current study demonstrated that gender-specific disparities in muscular fatty infiltration are attributed to mTOR/ULK1-mediated autophagy of FAPs. Decreased autophagic activity contributes to adipogenic differentiation in female FAPs after RCT. Moreover, metformin could enhance mTOR/ULK1-mediated autophagic processes of FAPs, thereby alleviating fatty infiltration and improving shoulder functionality after RCT. Therefore, metformin could be a promising noninvasive intervention to ameliorate muscular fatty infiltration of RCT.

## Graphical abstract

**Figure:**
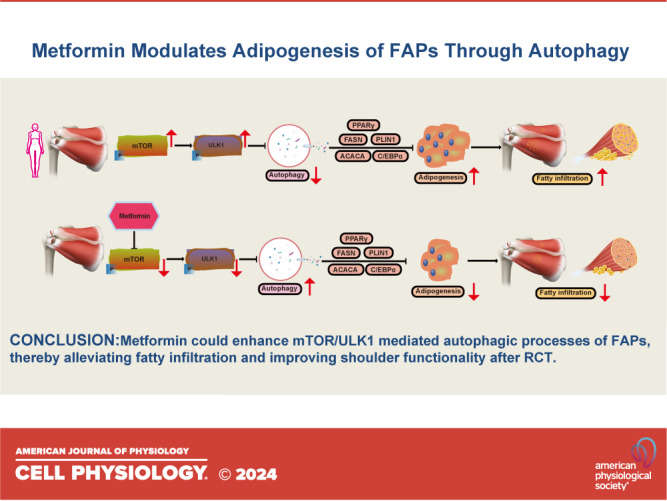

## INTRODUCTION

Rotator cuff tear (RCT) is a common musculoskeletal disease that frequently results in substantial pain and functional impairment (1). These pathological changes encompass muscle atrophy, fibrosis, and fatty infiltration (1, 2). The extent of muscular fatty infiltration significantly correlates with the patient’s prognosis (3–5). Even after surgical intervention, the progression of fatty infiltration within the rotator cuff continues to impair therapeutic outcomes and could potentially contribute to the recurrence of rotator cuff ruptures (1, 5). However, the mechanisms responsible for muscular fatty infiltration remain enigmatic. It is worth noting that female patients show a higher prevalence and greater severity of fatty infiltration when compared with male patients (3, 6). Nevertheless, the underlying causes of this phenomenon are unidentified. Thus, there is a compelling need to investigate the detailed mechanisms underlying this phenomenon, which could further improve the treatment strategy of RCT.

In recent years, fibro-adipogenic progenitors (FAPs) have gained recognition as key contributors in muscular fatty infiltration after RCT (1). Fibro-adipogenic progenitors in rotator cuff tear (RCT-FAPs) could experience a significant increase in their proliferative and adipogenic potential after RCT, resulting in fatty accumulation (7). Previous researches have proposed that the mechanisms behind fatty infiltration by RCT-FAPs may involve changes in localized signaling cascades, epigenetic modifications, baseline variations among FAP subgroups, inflammatory mediators, and complex interactions with immune cells (1). Nevertheless, the complete mechanisms governing fatty infiltration triggered by FAPs following RCT remain obscure. When considering the gender difference of muscular fatty infiltration, exploring the distinctions in characteristics of RCT-FAPs between different genders holds significant value.

Autophagy is a cellular process involving the degradation of organelles and cellular components, which emerges as a vital adaptation mechanism in response to novel environmental stresses (8). A growing body of evidence suggests that autophagy plays a crucial role in coordinating muscle cell remodeling after injuries or disease (9–11). Moreover, autophagy plays a vital role in adipogenic differentiation (12–16). Thus, autophagy within FAPs may potentially serve as a crucial factor in the context of RCT.

The primary objective of this study is to scrutinize gender-specific functional disparities of FAPs among patients with RCT, identify the association between excessive fat accumulation and autophagy, and formulate prospective strategies for mitigating the progression of fatty infiltration. Here, we found that RCT-FAPs from female patients exhibited pronounced fatty infiltration but reduced autophagy activity compared with male patients. Decreased mTOR/ULK1-mediated autophagic activity could contribute to adipogenic differentiation in FAPs. Moreover, metformin had the potential to mitigate fatty infiltration after RCT both in vivo and in vitro by enhancing mTOR/ULK1-mediated autophagic processes.

## MATERIALS AND METHODS

### Human Sample

The study was approved by the local institutional ethics committee (Approval No. XHEC-D-2022-129). From July 2022 to July 2024, we collected supraspinatus muscle samples from patients with or without RCT, as previously described (17). Informed consents have been obtained by all participants.

### Animals

All animal experiments received approval from animal care and use committee at local institution (Approval No. XHEC-F-2023-028). We housed 12-wk-old C57BL/6J female mice (Animal Model Research Center of Xinhua Hospital affiliated to Shanghai Jiao Tong University School of Medicine) and provided them with free access to water and a standard diet. The animal model for RCT was constructed according to established procedures (18, 19). In brief, a lateral skin incision was made at the shoulder to expose the supraspinatus and infraspinatus tendons, which inserted into the greater tuberosity. Both supraspinatus and infraspinatus tendons were then surgically detached from the humeral greater tuberosity to create the RCT model. Subsequently, the skin was closed without repairing the tendons.

### Isolation of Muscle FAPs

The digestion procedure for obtained muscle was performed as previously reported (20, 21). Muscle tissue was first finely dissected, then subjected to 1-h digestion with collagenase II (Worthington Biochemical, 700–800 U/mL, Cat. No. LS004177), followed by a 30-min digestion with a combination of collagenase II and enzymes (Life Technologies, 11 U/mL, Cat. No. 17105-041). The digested mixture underwent 10 passes through a 20-gauge needle and was subsequently filtered using a 40-µm cell strainer (BD Falcon, Cat. No. 352340). Cell preparation for flow cytometry was adapted from existing literature (19–24). For the isolation of human FAPs, we used Percp/cy5.5 anti-human CD31 (BioLegend, Cat. No. 30313), Pecy5 anti-human CD45 (BD Biosciences, Cat. No. 555484), BV421 anti-human CD56 (BioLegend, Cat. No. 562751), and APC anti-human CD34 (BioLegend, Cat. No. 343510). Subsequently, human CD31^−^/CD45^−^/CD56^−^/CD34^+^ FAPs were isolated via fluorescence-activated cell sorting using the BD Influx sorter.

### Cell Culture, Adipogenic Differentiation, and Treatment

Primary FAPs were cultured in α-MEM (Cellgro, Cat. No. 10-022-CV) containing 20% FBS (Yeasen, Cat. No. 40131ES76) and 1% penicillin-streptomycin (Gibco, Cat. No. 15140-122) at 37°C under 5% carbon dioxide. The adipogenic differentiation medium (ADM) comprised α-MEM (Cellgro, Cat. No. 10-022-CV) supplemented with 20% FBS (Yeasen, Cat. No. 40131ES76), 1% penicillin-streptomycin (Gibco, Cat. No. 15140-122), 1 μg/mL insulin (Sigma-Aldrich, Cat. No. I2643), 0.25 µM dexamethasone (Sigma-Aldrich, Cat. No. D4902), and 0.5 mM 3-isobutyl-1-methylxanthine (Sigma-Aldrich, Cat. No. I5879). In addition, 10 nM Bafilomycin A1 (MedChemExpress, Cat. No. HY-100558), 100 nM rapamycin (absin, Cat. No. abs810030), and 0.5 mM metformin (MedChemExpress, Cat. No. HY-B0627) were used in specific experiments. Only female FAPs were used in the experiments to confirm the relationship between autophagic activity and the ability of adipogenic differentiation.

### Immunohistology and Immunofluorescent Staining

Frozen sections or cultured cells were fixed in PBS containing 4% paraformaldehyde (Sigma-Aldrich, Cat. No. 30525) for 15 min, permeabilized in 0.5% Triton X-100 at room temperature for 15 min, and subsequently blocked with 1% BSA in PBS for 1 h (Beyotime Biotechnology, Cat. No. ST023). Subsequently, samples were incubated overnight at 4°C with anti-PDGFRα (Abcam, Cat. No. ab203491), anti-laminin (Abcam, Cat. No. ab44941), and anti-perilipin A/B (Millipore, Cat. No. P1873) antibodies. Alexa 488- or Alexa 594-conjugated anti-rat and anti-rabbit secondary antibodies (Invitrogen) were applied and incubated at room temperature for 1 h. DAPI (Vector Laboratories, Cat. No. H-1200) was used to stain cell nuclei, followed by mounting with an anti-fade reagent (Vector Laboratories, Cat. No. H-100), and imaging was performed using a Leica SP8 confocal microscope. Image analysis for all pictures was conducted using Image J software.

### Oil Red Staining

To assess lipid content, differentiated FAPs were fixed in 4% paraformaldehyde for 15 min. Afterward, a 15-min permeabilization step was carried out at room temperature using 0.5% Triton X-100, followed by a 10-min oil red (Solarbio, Cat. No. G1260) treatment. Finally, DAPI was used for nuclear staining and fluorescence imaging, and subsequent analysis was performed.

### Triglycerides Quantification Analysis

The degree of muscular fatty infiltration was assessed using a triglyceride colorimetric assay kit (Elabscience, Cat. No. E-BC-K261-M). In brief, the samples were homogenized in isopropanol and subsequently centrifuged at 10,000 *g* for 3 min. The resulting supernatant was collected, and triglyceride levels were quantified at an absorbance of 510 nm using a microplate reader.

### EdU Label

The assessment of cell proliferation capacity used the EdU assay kit (RiboBio, Cat. No. C10310-3) following the manufacturer’s guidelines. Cells underwent fixation in 4% PFA for 30 min, followed by a 10-min permeabilization step with 0.5% Triton X-100 at room temperature. Next, cells were stained using the Apollo reaction cocktail (RiboBio, Cat. No. C10310-3) along with DAPI. Finally, samples were mounted with an antifade reagent, and subsequent imaging analysis was performed.

### Autolysosome Staining

DALGreen (Dojindo, Cat. No. D675) was used to stain autolysosomes for the assessment of cellular autophagic flux. After a 30-min incubation of working solution, samples underwent two PBS washes and were further processed according to the experimental needs. Imaging analysis of arbitrary units (AU) was performed by using a Leica SP8 confocal microscope.

### Gene Expression Analysis

We used the TRIzol reagent (Invitrogen, Cat. No. 15596-018) for the extraction of total RNA. Subsequently, reverse transcription was carried out at 42°C for 60 min using the MuLV reverse transcriptase (NEB, Cat. No. M0253L). Following it, quantitative real-time polymerase chain reaction (RT-qPCR) was performed using the Universal SYBR Green Fast qPCR Mix (RK21203) in the Bio-Rad real-time PCR system (CFX Maestro) with GAPDH serving as the internal reference. The detailed RT-qPCR primers are listed as following:

GAPDH forward 5′- CAAGGCTGAGAACGGGAAGC-3′ and reverse 5′- AGGGGGCAGAGATGATGACC-3′; ACACA forward 5′- ATCTTGAGGGCTAGGTCTTTTT-3′ and reverse 5′ AGAGTGCTGGTTCAGCTCC-3′; FASN forward 5′ AAGGACCTGTCTAGGTTTGATGC-3′ and reverse 5′ TGGCTTCATAGGTGACTTCCA -3′; peroxisome proliferator-activated receptor γ (PPARγ) forward 5′- CCAGAAGCCTGCATTTCTGC-3′ and reverse 5′- CACGGAGCTGATCCCAAAGT-3′; PLIN1 forward 5′- TGTGCAATGCCTATGAGAAGG-3′ and reverse 5′- AGGGCGGGGATCTTTTCCT-3′; C/EBPα forward 5′- TATAGGCTGGGCTTCCCCTT-3′ and reverse 5′- AGCTTTCTGGTGTGACTCGG-3′.

### Western Blotting

The protein samples underwent separation via sodium dodecyl sulfate-polyacrylamide gel electrophoresis (SDS-PAGE) and were subsequently transferred onto polyvinylidene fluoride (PVDF) membranes. These membranes were blocked with 5% BSA in TBST for 1 h and then subjected to overnight incubation at 4°C on a shaker with primary antibodies targeting LC3B (ABclonal, Cat. No. A19665), mTOR (Proteintech, Cat. No. 66888-1-lg), phospho-mTOR (Proteintech, Cat. No. 67778-1-lg), ULK1 (CST, Cat. No. 8054), phospho-ULK1 (CST, Cat. No. 14202), and GAPDH (CST, Cat. No. 2118L). Following a 1-h incubation with the corresponding secondary antibodies, chemiluminescence was used for target protein imaging, and subsequent quantification analysis was performed using Image J.

### Gene Overexpression

In brief, 2 μL of Lipofectamine 2000 (Invitrogen, Cat. No. 11668019) and 1 μg of the overexpression plasmid (MIAOLING BIOLOGY, Cat. No. P39375) were added into 100 μL of Opti-MEM I Reduced Serum Medium (Thermo Fisher, Cat. No. 31985062), respectively. After mixing these components and incubating them at room temperature for 15 min, we introduced the DNA-lipid complex into the cultured FAPs. Subsequent experiments were performed 48 h after transfection.

### Gait Analysis

We used the Noldus CatWalk system to quantify the stride length, stance width, and paw surface area of the experimental mice, aiming to assess their shoulder abduction, load-bearing capacity, and pain level, as previously described (19, 25). The mice were placed within an 85-cm-long and 8.5-cm-wide corridor, where they could move freely. The movements were recorded by focusing on tracks marked by consistent and uninterrupted trajectories. A minimum of five walking paths were documented for each mouse.

### Treadmill Test

Functional testing was performed by using a treadmill apparatus (ZII-PT/5S). Before formal testing, mice underwent a 2-day adaptation training period. During the exhaustion test, we initiated the treadmill at 10 m/min with a 15° incline. The speed was then incremented by 2 m/min every 2 min until reaching a maximum of 20 m/min. Exhaustion was determined if the mice kept still withstanding three manual pushes, in conjunction with their behavior in the new environment. Subsequently, exercise time and distance were documented.

### Bulk RNA Sequencing and Analysis

After acquiring purified mRNA, we proceeded to create RNA sequencing (RNA-Seq) libraries using the NEBNext Ultra RNA Library Prep Kit for Illumina (New England Biolabs, Cat. No. E7530L). Subsequently, a cDNA library was fashioned, featuring mean inserts of 300 bp, using a nonstranded library preparation method. Paired-end sequencing was conducted, with a 2X 150 bp read length, using the NovaSeq 6000 sequencer. To ensure data quality, quality control was performed on the raw paired-end reads using SeqPrep (https://github.com/jstjohn/SeqPrep) and Sickle (https://github.com/najoshi/sickle).

Next, the clean paired-end reads were aligned to the reference genome GRCh38.98 using HISAT2. RSEM (http://deweylab.biostat.wisc.edu/rsem/) was used to quantify gene abundances (26). The differential expression analysis was conducted using DEGseq. It was considered as significantly differentially expressed genes with a *P* value of <0.05 (27).

For further insights, Gene Ontology (GO) and Kyoto Encyclopedia of Genes and Genomes (KEGG) analyses were performed. These analyses were carried out using Goatools (https://github.com/tanghaibao/Goatools) and KOBAS (http://kobas.cbi.pku.edu.cn/home.do), respectively (28).

### Statistical Analysis

Statistical analysis was carried out with GraphPad Prism9 (GraphPad), and the results were expressed as means ± standard deviation. Data comparisons were performed using *t*-tests and one-way or two-way analysis of variance (ANOVA). Statistical significance was determined at *P* < 0.05. All the analyses were performed in a blind manner, and each experiment consisted of at least three biological replicates and three technical replicates.

## RESULTS

### There Was More Muscular Fatty Infiltration in Female Patients with Rotator Cuff Tear When Compared with Males

**Figure 1.**
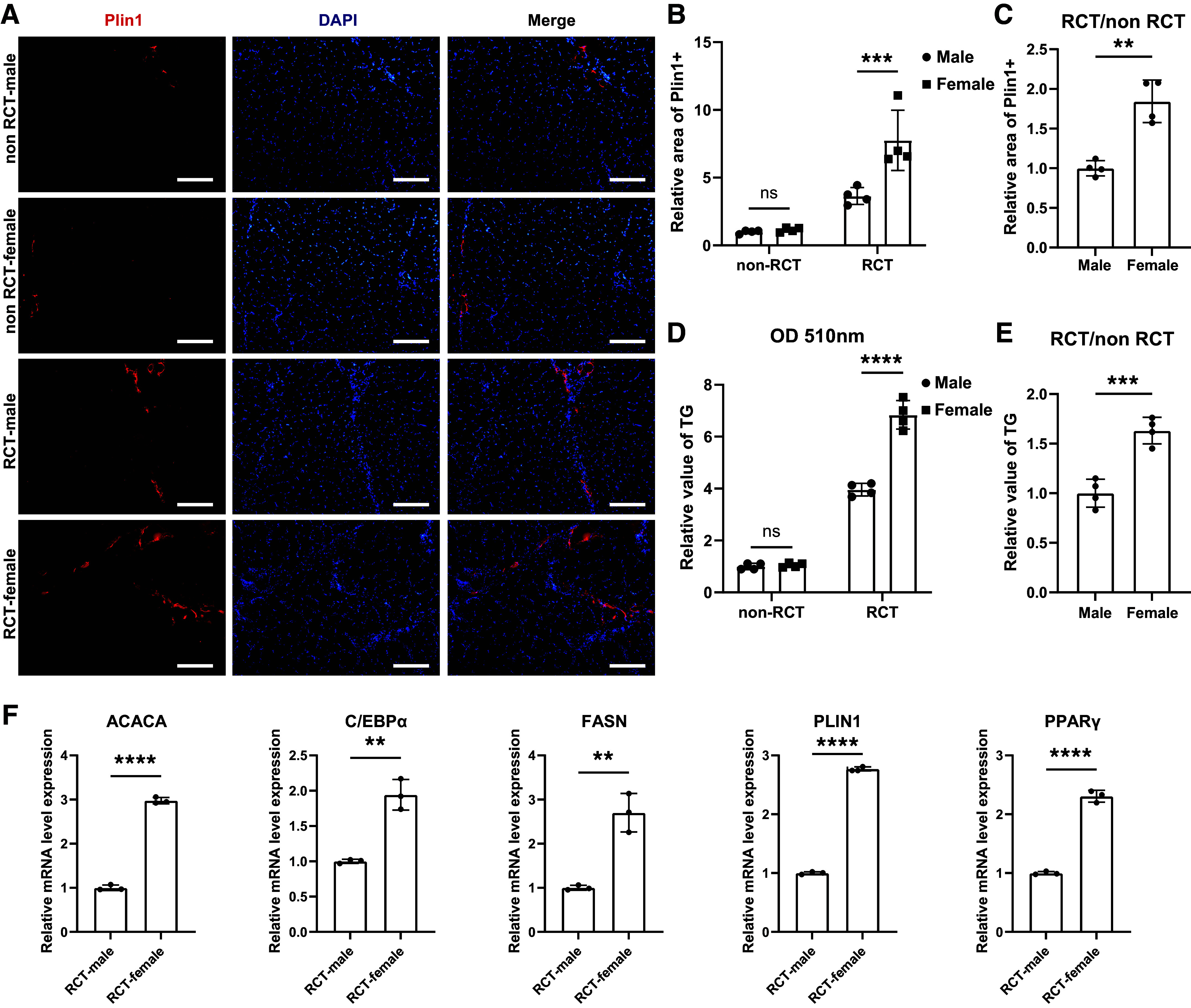
There was more muscular fatty infiltration in female patients with rotator cuff tear when compared with males. *A*–*C*: immunofluorescence staining and quantification analysis of lipid droplets in the supraspinatus muscles of female and male patients with or without RCT (*n* = 4 patients/group). Scale bar = 150 μm. *D* and *E*: quantification measurement of triglycerides in the supraspinatus muscle between female and male patients with or without RCT (*n* = 4 patients/group). *F*: the relative mRNA expression of adipogenesis-related genes in supraspinatus muscles of female and male patients after RCT (*n* = 3 patients/group). Data were shown as means ± SD. RCT, rotator cuff tear; TG, triglyceride. **P < 0.01; ***P < 0.001; ****P < 0.0001.

To investigate gender differences in muscular fatty infiltration following RCT, we first obtained supraspinatus muscle samples from both female and male patients. There were no statistical differences in clinical characteristics including age, BMI, glucose, and tear size between women and men (Supplemental Table S1). Histological sections of supraspinatus muscles from female patients exhibited no statistically significant differences in noninjured group, but they displayed more extensive muscular fat accumulation in RCT group, as evidenced by immunofluorescent staining of Plin1 (Fig. 1, *A*–*C*). Moreover, triglyceride level was also elevated in supraspinatus muscles from female patients (Fig. 1, *D* and *E*). Consistently, RT-qPCR results also revealed upregulated expression of key adipogenesis-related genes in female supraspinatus muscles with RCT (Fig. 1*F*). In summary, these findings indicated that female patients experienced more pronounced fatty infiltration in their supraspinatus muscles following RCT when compared with males.

### Increased Adipogenic Differentiation Capacity and Decreased Autophagy Activity Were Identified in Female RCT-FAPs

Given the pivotal role of FAPs in muscular fatty infiltration after RCT (1), we next investigated the differences of FAPs between males and females. Immunofluorescence staining of key marker PDGFRα demonstrated that FAPs existed in supraspinatus muscle from both female and male patients (Supplemental Fig. S1, *A* and *B*). Then human RCT-FAPs from supraspinatus muscle were purified by fluorescence-activated cell sorting system (Supplemental Fig. S1*C*). The immunofluorescence staining of PDGFRα revealed that more than 90% of purified cells were FAPs (Supplemental Fig. S1, *D* and *E*). Thus, highly purified RCT-FAPs were obtained.

**Figure 2.**
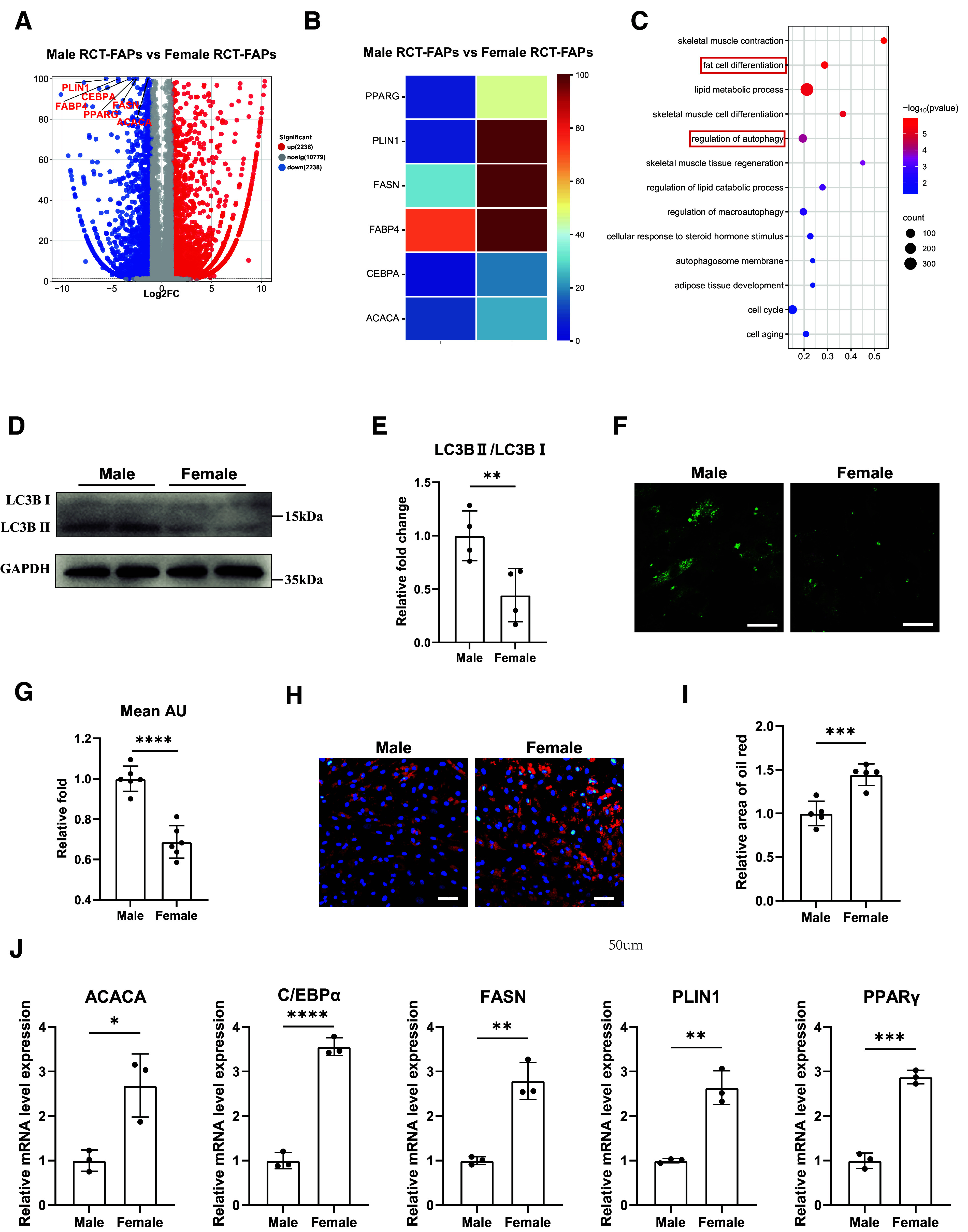
Increased adipogenic differentiation capacity and decreased autophagy activity were identified in female RCT-FAPs. *A*: volcano plot of differentially expressed genes in RCT-FAPs between female and male patients. Adipogenesis-related genes were highlighted. *B*: heatmap illustrating adipogenic-related gene expression profiles in RCT-FAPs between female and male patients. *C*: bubble chart of GO analysis of differentially expressed genes in RCT-FAPs between female and male patients. *D* and *E*: Western blotting and quantification analysis of LC3B-I, LC3B-II, and GAPDH in freshly isolated RCT-FAPs from different genders (*n* = 4 mice/group). *F* and *G*: DALGreen staining and quantification analysis of freshly isolated RCT-FAPs from different genders (*n* = 6/condition). Scale bar = 25 μm. *H* and *I*: oil red staining and quantification analysis of RCT-FAPs between different genders after induction with adipogenic differentiation medium for 10 days (*n* = 5/condition). Scale bar = 50 μm. *J*: the relative mRNA expression of adipogenesis-related genes in RCT-FAPs between female and male after 10-day adipogenic differentiation (*n* = 3 patients/group). Data were shown as means ± SD. GO, Gene Ontology; RCT-FAP, fibro-adipogenic progenitors in rotator cuff tear. *P < 0.05; **P < 0.01; ***P < 0.001; ****P < 0.0001.

We next performed RNA-Seq analysis to compare the expression profiles of freshly isolated RCT-FAPs from female and male patients (Fig. 2, *A*–*C*). RNA-Seq demonstrated elevated expression of adipogenesis-related genes (PPARγ, PLIN1, FASN, FABP4, CEBPα, and ACACA) in female RCT-FAPs than males (Fig. 2, *A* and *B*). GO enrichment analysis of the differentially expressed genes enriched key terms including fat cell differentiation and regulation of autophagy (Fig. 2*C*). To confirm the results of RNA-Seq, the autophagy activity and adipogenic differentiation ability of RCT-FAPs were evaluated. The LC3-II/LC3-I ratio is a widely used metric for assessing autophagic levels (16, 29). Western blot results revealed significantly lower LC3-II/LC3-I ratio in female RCT-FAPs when compared with males, verifying reduced autophagic activity in female RCT-FAPs (Fig. 2, *D* and *E*). The autophagosome staining also demonstrated a significantly reduced fluorescence intensity of autophagosomes in female RCT-FAPs (Fig. 2, *F* and *G*). For adipogenic differentiation ability evaluation, both oil red staining and RT-qPCR analysis revealed enhanced adipogenic differentiation potential of female RCT-FAPs (Fig. 2, *H*–*J*). Taken together, these results highlighted that female RCT-FAPs exhibited a lower autophagy activity but enhanced adipogenic differentiation potential.

### mTOR/ULK1 Pathway-Mediated Autophagy Inhibited Adipogenic Differentiation in FAPs

**Figure 3.**
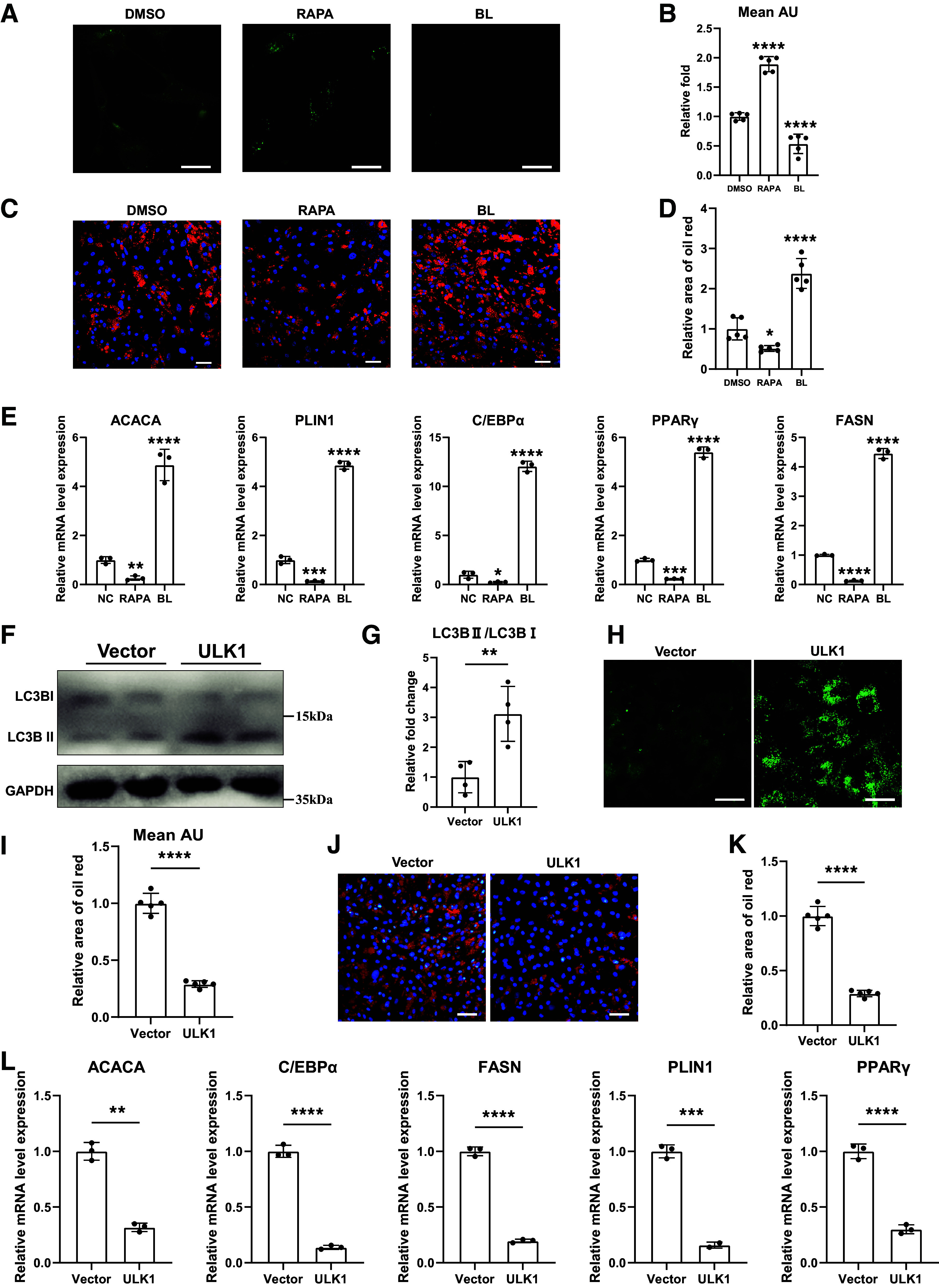
mTOR/ULK1 pathway-mediated autophagy inhibited adipogenic differentiation in FAPs. *A* and *B*: Autolysosome staining and quantification analysis of FAPs with 2-h treatment of dimethyl sulfoxide (DMSO), autophagy agonist rapamycin (RAPA), or autophagy inhibitor bafilomycin (BL), respectively (n = 5/condition). Scale bar = 25 μm. *C* and *D*: oil red staining and quantification analysis of lipid droplet formation of FAPs after 10-day adipogenic differentiation with treatment of dimethyl sulfoxide (DMSO), autophagy agonist rapamycin (RAPA), or autophagy inhibitor bafilomycin (BL) (*n* = 5/condition). Scale bar = 50 μm. *E*: the relative mRNA expression of adipogenesis-related genes in FAPs after 10-day adipogenic differentiation with treatments of dimethyl sulfoxide (DMSO), autophagy agonist rapamycin (RAPA), or autophagy inhibitor bafilomycin (BL), respectively (*n* = 3/condition). *F* and *G*: the protein levels and quantification analysis of LC3-I and LC3-II after transfection with plasmid overexpressing ULK1 in FAPs (*n* = 4/condition). *H* and *I*: autolysosome staining and quantification analysis of FAPs after transfection of ULK1 overexpression plasmid (*n* = 5/ condition). Scale bar = 25 μm. *J* and *K*: oil red staining and quantification analysis of FAPs between vector group and ULK1 overexpression group after 10 days of adipogenic differentiation (*n* = 5/condition). Scale bar = 50 μm. *L*: the relative mRNA expression of adipogenesis-related genes in adipogenically differentiated FAPs between vector group and ULK1 overexpression group (*n* = 3/condition). Data were shown as means ± SD. FAP, fibro-adipogenic progenitor. Relative area of oil red *P < 0.05; **P < 0.01; ***P < 0.001; ****P < 0.0001.

Since earlier studies have shown pivotal role of autophagy in adipogenesis of adipocytes (12, 13, 30), we next investigated the link between autophagy and adipogenesis in FAPs. The FAPs were adipogenically induced with treatment of DMSO, autophagy agonist rapamycin, or the autophagy inhibitor bafilomycin. DALGreen staining confirmed that rapamycin treatment significantly increased autophagic levels, whereas bafilomycin treatment reduced autophagic levels in FAPs (Fig. 3, *A* and *B*). Subsequently, oil red staining of adipogenically differentiated FAPs showed significantly decreased fatty infiltration after activating autophagy, whereas inhibiting autophagic activity increased the fat accumulation (Fig. 3, *C* and *D*). Furthermore, RT-qPCR results also verified inverse relationship between autophagy and expression level of adipogenesis genes (Fig. 3*E*). These data indicated that autophagy inhibited adipogenic differentiation potential of FAPs.

Then underlying mechanism of how autophagy was regulated for adipogenesis of FAPs was investigated. We noticed that the mTOR signaling was enriched in KEGG analysis of differentially expressed genes between female and male RCT-FAPs (Supplemental Fig. S2*A*). It has been confirmed that mTOR signaling pathway plays a pivotal role in regulating autophagy (29, 31). It was reported that the mTOR/ULK1 signaling could regulate gender-based differences in autophagy and adipogenesis within adipocytes (32). Nevertheless, the impact of the mTOR/ULK1-mediated autophagic activity in FAPs remains unexplored. Thus, we next investigated whether mTOR/ULK1 pathway regulated the gender difference of autophagy and adipogenesis in RCT-FAPs. The protein level of phospho-mTOR/total mTOR and phospho-ULK1/total ULK1 significantly decreased in male RCT-FAPs when compared with female RCT-FAPs (Supplemental Fig. S2, *B* and *C*), indicating activated mTOR/ULK1 pathway in female RCT-FAPs. To investigate the effects of ULK1, ULK1 overexpression was performed by transfecting overexpression plasmid in FAPs (Supplemental Fig. S2, *D* and *E*). After ULK1 overexpression, the LC3-II/LC3-I ratio was significantly increased (Fig. 3, *F* and *G*). Consistently, DALGreen staining also showed increased autophagosome levels after ULK1 overexpression (Fig. 3, *H* and *I*). Oil red staining and RT-qPCR demonstrated decreased fat accumulation in differentiated FAPs after ULK1 overexpression (Fig. 3, *J*–*L*). Combined, these data indicated that activated mTOR/ULK1 pathway in female RCT-FAPs inhibited autophagy activity in FAPs and then increased the adipogenic differentiation potential.

### Metformin Alleviated Muscular Fatty Infiltration in RCT Mice via Inhibition of mTOR/ULK1 Pathway

**Figure 4.**
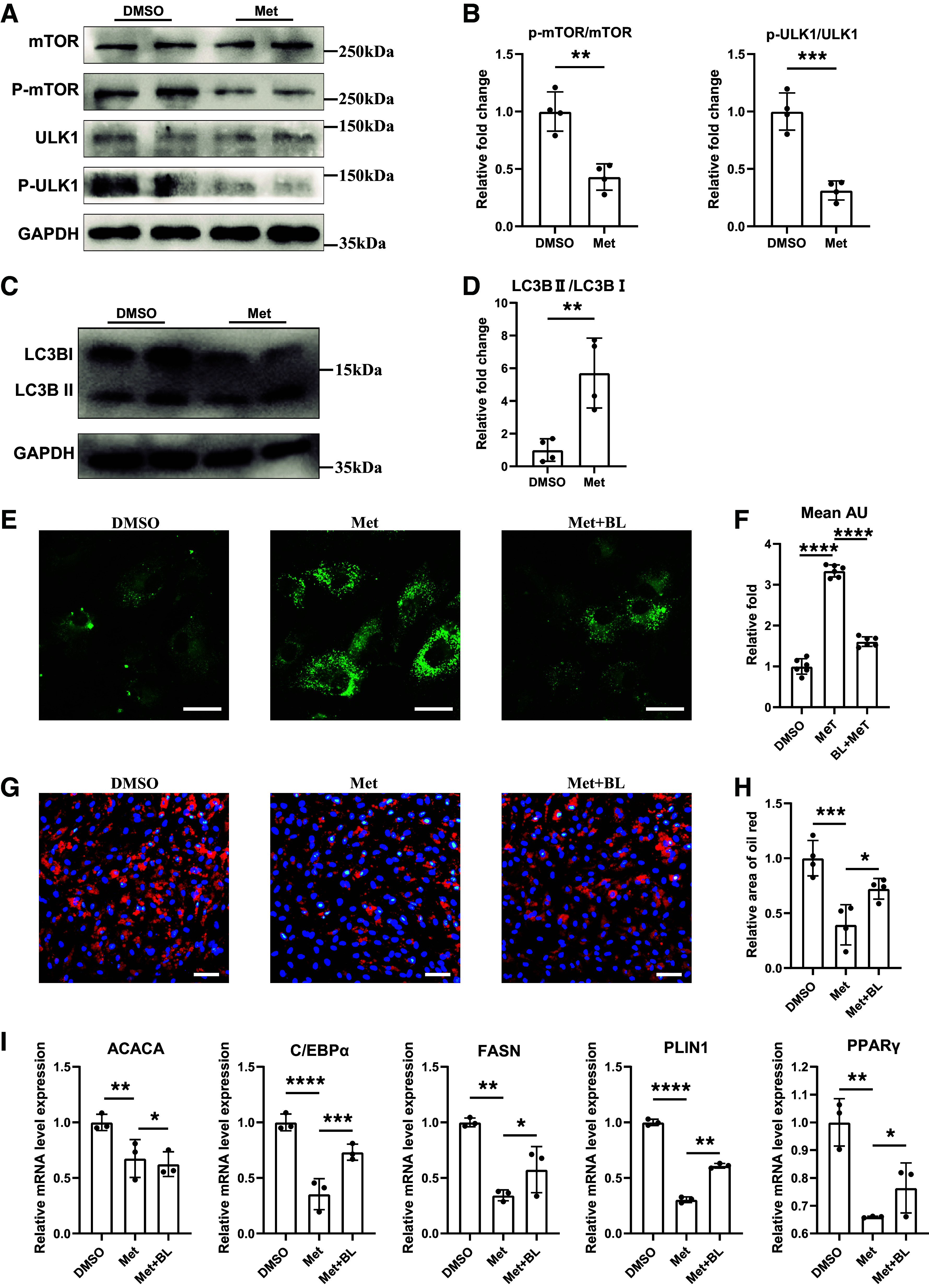
Metformin increased autophagy and suppressed adipogenic differentiation of FAPs via mTOR/ULK1 pathway. *A* and *B*: The protein levels and quantification analysis of mTOR, phospho-mTOR, ULK1, phospho-ULK1, and GAPDH in FAPs with a 2-h treatment of dimethyl sulfoxide (DMSO) or metformin (Met) (*n* = 4/condition). *C* and *D*: the protein levels and quantification analysis of LC3-I, LC3-II, and GAPDH in FAPs with a 2-h treatment of DMSO (DMSO) or metformin (Met) (*n* = 4/condition). *E* and *F*: DALGreen staining and quantification analysis of FAPs with treatments of dimethyl sulfoxide (DMSO), metformin (Met), and metformin combined with autophagy inhibitor bafilomycin (Met + BL), respectively (*n* = 6/condition). Scale bar = 25 μm. *G* and *H*: oil red staining and quantification analysis of FAPs in the dimethyl sulfoxide group (DMSO), metformin group (Met), and metformin combined with autophagy inhibitor bafilomycin group (Met + BL) after 10-day induction of adipogenesis (*n* = 5/condition). Scale bar = 50 μm. *I*: the relative mRNA expression of adipogenesis-related genes of FAPs in the dimethyl sulfoxide group (DMSO), metformin group (Met), and metformin combined with autophagy inhibitor bafilomycin group (Met + BL) after 10-day induction of adipogenesis (*n* = 3/condition). Data were shown as means ± SD. FAP, fibro-adipogenic progenitor. *P < 0.05; **P < 0.01; ***P < 0.001; ****P < 0.0001.

We next tried to find the molecules capable of increasing autophagic activity and suppressing excessive adipogenic differentiation potential of female RCT-FAPs. Since metformin is able to regulate mTOR/ULK1-mediated autophagy in various cell types (33), we explored whether metformin could also regulate mTOR/ULK1 pathway and thus enhance autophagy and suppress adipogenic differentiation of FAPs. After metformin treatment, the Western blot results demonstrated that metformin could significantly inhibit mTOR/ULK1 pathway in FAPs (Fig. 4, *A* and *B*). In addition, metformin also increased LC3-II/LC3-I ratio and autolysosomes (Fig. 4, *C*–*F*), indicating activated autophagy level by metformin. Consistently, the increased autophagy level by metformin also inhibited the adipogenic differentiation potential of FAPs (Fig. 4, *G*–*I*). However, when treating FAPs with metformin and autophagy inhibitor bafilomycin, the inhibition effect of adipogenesis by metformin was counteracted (Fig. 4, *G*–*I*). Taking together, these data illustrated that metformin could increase mTOR/ULK1-mediated autophagic activity and thus suppress adipogenic differentiation potential of FAPs.

### Metformin Reduced Fatty Infiltration and Improved Shoulder Function

Since metformin showed a promising effect to counteract excessive adipogenic differentiation ability of FAPs, we then explored whether metformin could impede fat infiltration and improve shoulder function in RCT model.

**Figure 5.**
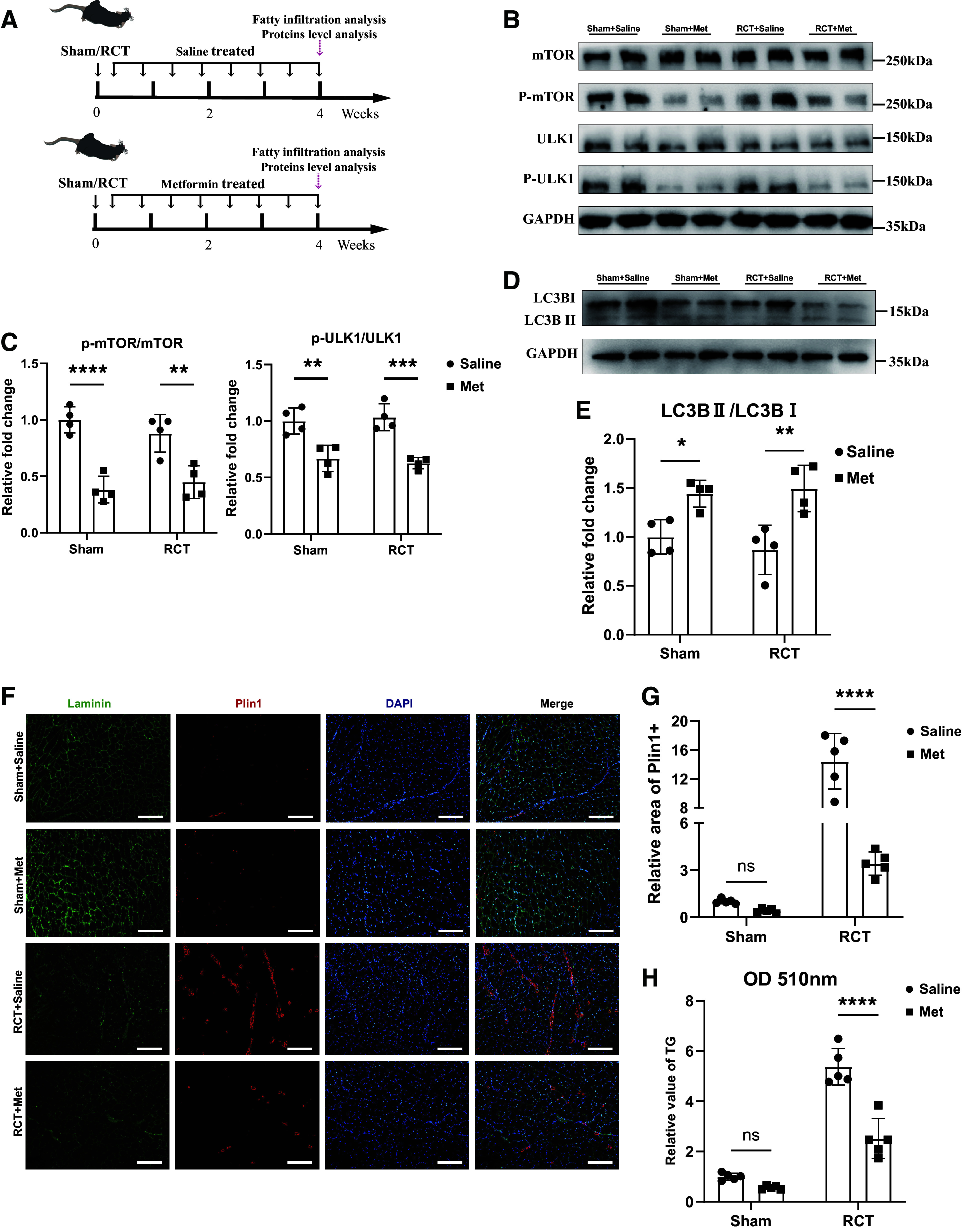
Metformin alleviated muscular fatty infiltration in RCT mice via inhibition of mTOR/ULK1 pathway. *A*: Schematic diagram of the muscular fatty infiltration and protein levels evaluation after RCT. *B* and *C*: protein expression and quantification analysis of mTOR, phospho-mTOR, ULK1, phospho-ULK1, and GAPDH in freshly isolated FAPs in the sham group with saline (Sham + Saline), sham group with metformin (Sham + Met), RCT group with saline (RCT + Saline), and RCT group with metformin (RCT + Met) (*n* = 4 mice/group). *D* and *E*: protein expression and quantification analysis of LC3B-I, LC3B-II, and GAPDH in freshly isolated FAPs in sham group with saline (Sham + Saline), sham group with metformin (Sham + Met), RCT group with saline (RCT + Saline), and RCT group with metformin group (RCT + Met) (*n* = 4 mice/group). *F* and *G*: immunofluorescence staining and quantification measurement of muscular fatty infiltration in sham group with saline (Sham + Saline), sham group with metformin (Sham + Met), RCT group with saline (RCT + Saline), and RCT group with metformin group (RCT + Met) (*n* = 5 mice/group). Scale bar = 150 μm. *H*: quantification analysis of triglycerides in the supraspinatus muscles of RCT mice in sham group with saline (Sham + Saline), sham group with metformin (Sham + Met), RCT group with saline (RCT + Saline), and RCT group with metformin group (RCT + Met) (*n* = 5 mice/group). Data were shown as means ± SD. FAP, fibro-adipogenic progenitor; RCT, rotator cuff tear. *P < 0.05; **P < 0.01; ***P < 0.001; ****P < 0.0001. All RCT mice were female.

Upon establishing the RCT model, we administered metformin via drinking water at a dosage of 100 mg/kg for 4 wk. After 4 wk treatment, there were no statistically significant differences in food intake, body weight, and blood glucose levels in metformin treatment group when compared with saline-treated group (Supplemental Fig. S3). After completing the treatment, we euthanized the mice and harvested their supraspinatus muscles for subsequent assessments (Fig. 5*A*). Fresh FAPs were isolated to investigate the effects of oral metformin to FAPs. The Western blot results revealed that oral metformin treatment inhibited mTOR/ULK1 pathway of FAPs (Fig. 5, *B* and *C*). Furthermore, oral metformin also increased the ratio of LC3-II/LC3-I in FAPs, indicating activated autophagy level (Fig. 5, *D* and *E*). After metformin treatment, both immunohistochemistry and triglyceride quantification showed decreased muscular fatty infiltration (Fig. 5, *F*–*H*). These data demonstrated that oral metformin also worked in vivo to increase mTOR/ULK1-mediated autophagy, thus contributing to alleviating excessive fatty infiltration after RCT.

**Figure 6.**
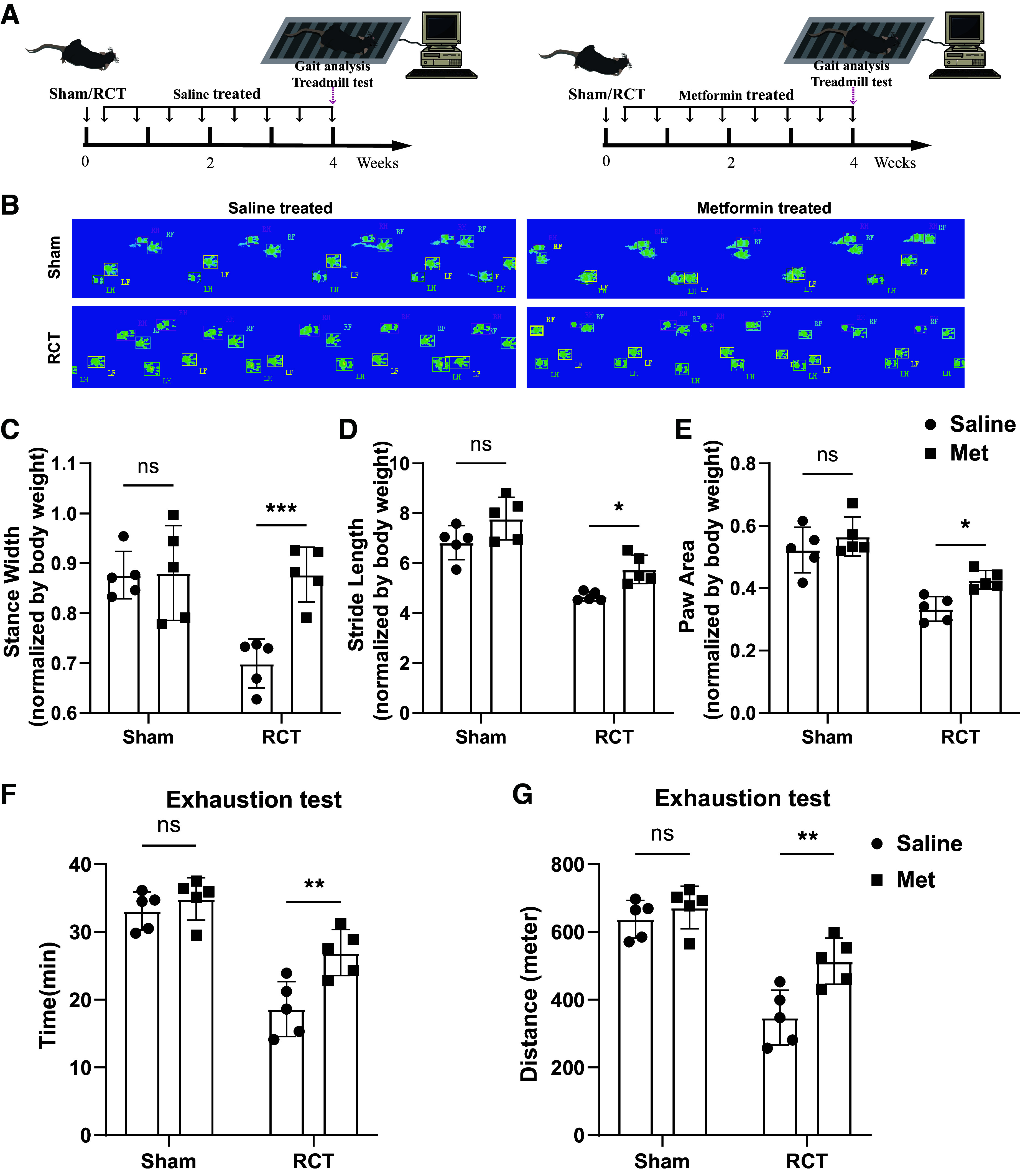
Metformin improved shoulder function after rotator cuff tear. *A*: schematic diagram of the in vivo shoulder function evaluation after RCT. *B*–*E*: gait analysis and associated parameters (stance width, stride length, and paw area) of sham and RCT mice with or without metformin treatment while correcting for the body weight (*n* = 5 mice/group). *F* and *G*: treadmill tests of sham and RCT mice with or without metformin treatment (*n* = 5 mice/group). Data were shown as means ± SD. RCT, rotator cuff tear. *P < 0.05; **P < 0.01; *** indicated *P* < 0.001. All RCT mice were female.

To further evaluate the shoulder function of RCT model after metformin treatment, gait analysis and treadmill tests were conducted (Fig. 6*A*). In gait analysis assay, parameters such as stride length, stance width, and paw contact area were measured to assess shoulder joint abduction, weight-bearing capacity, and pain levels. No statistically significant differences were observed between the sham group with or without metformin treatment. However, all these parameters significantly improved in RCT metformin treatment group (Fig. 6, *B*–*E*). In addition, running time and distance in treadmill tests were recorded to evaluate mice shoulder functional performance. Similarly, RCT mice treated with metformin also exhibited better performance in running distance and time when compared with the saline-treated group (Fig. 6, *F* and *G*). Combined, these data demonstrated that metformin could be a potential treatment strategy to alleviate fatty infiltration and improve shoulder function after RCT.

## DISCUSSION

**Figure 7.**
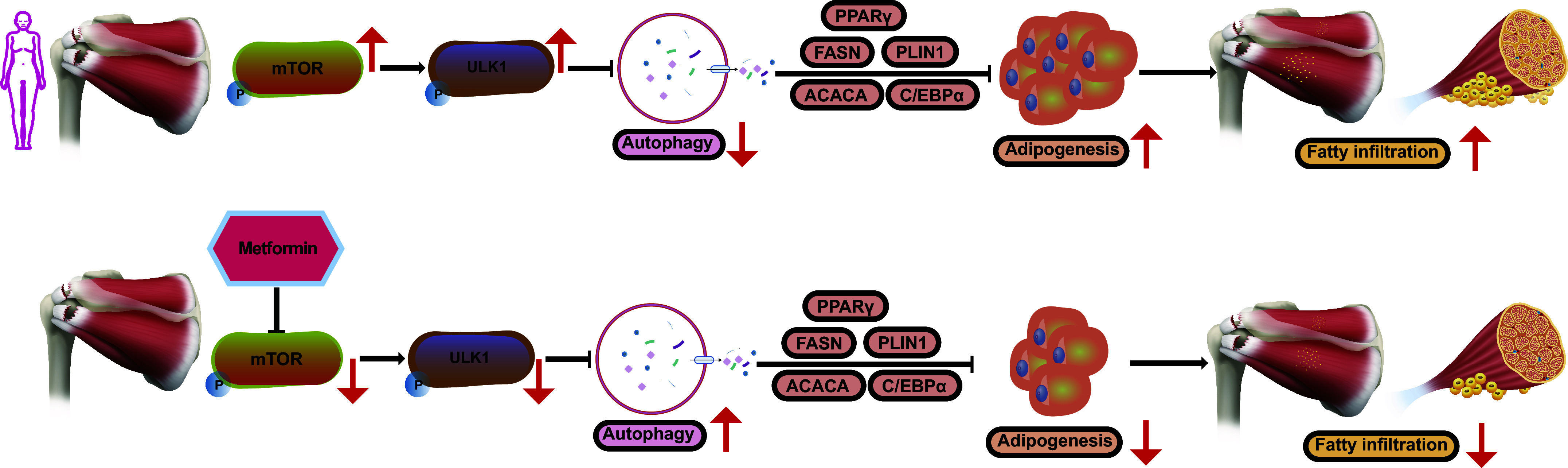
Schematic diagram of mTOR/ULK1 pathway-mediated autophagy and adipogenesis in FAPs. In female patients with RCT, increased mTOR/ULK1signaling inhibits autophagic activity of FAPs, which contributes to its excessive adipogenic differentiation and thus leads to gender-based variations in muscular fatty infiltration. However, metformin could activate mTOR/ULK1-mediated autophagy of female RCT-FAPs and thus alleviate the progression of muscular fatty infiltration. FAP, fibro-adipogenic progenitor; RCT, rotator cuff tear.

Our investigation unveiled a gender discrepancy in the adipogenic differentiation capabilities of RCT-FAPs, potentially arising from variations in autophagic activity. Female RCT-FAPs exhibited decreased autophagic activity, which resulted in an enhanced adipogenesis ability when compared with males. Metformin could enhance mTOR/ULK1-mediated autophagic processes of FAPs, thereby alleviating fatty infiltration and improving shoulder functionality after RCT (Fig. 7).

Prior studies have documented gender-based variations in fatty infiltration stemming from RCT (3, 6). Females manifest an elevated odds and greater severity of fatty infiltration in contrast with males (3, 6). Prior studies have documented variations in muscle characteristics between males and females. There was greater abundance of type I muscle fibers, improved capillary supply, and heightened insulin sensitivity in female skeletal muscles (34). However, there are few studies concentrating on why females undergo more severe fat infiltration after RCT. Thus, the primary purpose of current study is to elucidate the detailed mechanism of different adipogenesis status between males and females after RCT, which could provide a novel perspective for treating RCT.

Since FAPs act as one of the primary contributors of muscular fatty infiltration after RCT, numerous investigations have focused on regulating the adipogenic differentiation, proliferation, and abundance of FAPs for therapeutic purposes (19, 35, 36). Shirasawa et al. (35) used an RARγ agonist to attenuate the adipogenic differentiation of FAPs, consequently reducing fatty infiltration in an RCT mouse model. In addition, PDGFR signaling pathway inhibitor imatinib was demonstrated to decrease fat accumulation after RCT (36). Furthermore, Lemos et al. (37) found that fatty infiltration could be mitigated by activating the TNF signaling pathway and inducing apoptosis of FAPs. Thus, FAPs could be a promising target to treat excessive muscular fat infiltration after RCT.

One of the primary findings in current study is that gender variations in fatty infiltration after RCT could be caused by different degrees of autophagy activity. Autophagy serves the purpose of degrading and recycling impaired and senescent proteins/organelles, thereby upholding cellular equilibrium (29). Previous studies have underscored that certain gender-specific phenotypes could be orchestrated by autophagy in skeletal muscle (38, 39). Davegårdh et al. (38) discovered gender effects on DNA methylation, gene expression, and autophagic levels in human skeletal muscle myoblasts and myotubes. Meyer et al. (39) have highlighted the significant role of autophagy in the gender-specific nature of muscle atrophy induced by tendonectomy. Thus, autophagy is closely associated with muscle regeneration and remodeling (9–11). However, there is a dearth of knowledge regarding the effects of autophagy on the excessive adipogenesis of female muscle after RCT. FAPs are a distinct muscle stem cell population that have been regarded as the primary instigators of muscular fatty infiltration after RCT (1). It is also worth noting that autophagy is deemed indispensable in adipogenesis (12, 13, 30) and could mediate gender differences in visceral fat (32). These studies indicated potential role of different degrees of autophagy activity of FAPs between male and female after RCT. Expectedly, the current study found that female patients exhibited lower autophagic activity in RCT-FAPs, resulting in a greater adipogenic differentiation ability when compared with males.

Our findings revealed that metformin could reinstate autophagic activity and alleviate fat infiltration via the mTOR/ULK1 pathway. Metformin is a commonly prescribed medication for diabetes. Recent studies have shown its ability to stimulate autophagy and reduce fatty infiltration (16, 24, 33, 40). It could ameliorate hepatic steatosis through restoring autophagy (16). It also inhibits the adipogenesis of mesenchymal stem cells (40). Moreover, Farup et al. (24) observed that metformin alleviated the adipogenesis of FAPs from patients with type 2 diabetes mellitus. Thus, metformin was used to explore potential effects on alleviating excessive adipogenesis of FAPs after RCT. Although there are currently various treatment strategies for RCT, potential drawbacks warrant consideration. For example, physical therapy can alleviate pain and improve functionality to some extent, but it is time-consuming and does not hinder the progression of fat infiltration (41). In addition, the surgical repair for RCT imposes substantial economic burden and long recovery period, whereas the retear rate is closely associated with fatty infiltration (1, 5, 41). Therefore, there is a necessity to explore a more effective and economically feasible treatment approach. Interestingly, metformin has exhibited the capacity to diminish fatty infiltration and augment shoulder joint function in RCT model. Thus, the current study offered a new perspective on RCT treatment.

There are some limitations in the current research. First, our study exclusively focused on the autophagic pathway involving mTOR/ULK1 and the alterations in LC3-II/LC3-I, without delving into other key autophagy-related proteins such as beclin 1, Atg5, Atg7, and Atg12-Atg5 conjugate. In addition, the specific mechanisms governing how autophagy modulates the adipogenic differentiation of FAPs remain enigmatic. Thus, further researches are imperative for elucidating the detailed mechanisms of how autophagy governs the adipogenic differentiation of FAPs. Furthermore, differences in the shoulder anatomy and biomechanics between mouse and human might limit translational relevance of current study to some extent. Therefore, further clinical trials are needed to clarify the utilization, safety, and viability of metformin in addressing fat infiltration after RCT.

## ETHICAL APPROVAL

The study was approved by local ethical Committee (Approval No. XHEC-F-2023-028 and Approval No. XHEC-D-2022-129).

## DATA AVAILABILITY

The supporting data for the findings in this study can be obtained from the corresponding author upon a reasonable request.

## SUPPLEMENTAL DATA

10.6084/m9.figshare.24999071 Supplemental Figs. S1–S3 and Table S1: https://10.6084/m9.figshare.24999071.

## GRANTS

The sponsorships were obtained from Natural Science Foundation of China (No. 82372384), National Science Fund for Young Scholars (No. 82302657), and science and technology planning project of Linpin (LPWJ2023-02-27).

## DISCLOSURES

No conflicts of interest, financial or otherwise, are declared by the authors.

## AUTHOR CONTRIBUTIONS

Hao Zhou, X.L., X.S., S.F., and S.Z. conceived and designed research; Hao Zhou performed experiments; S.Z. analyzed data; X.L. interpreted results of experiments; X.L. prepared figures; Hao Zhou, X.L., and X.S. drafted manuscript; S.F., Han Zhou, H.C., H.Y., Z.W., R.W., X.S., and J.W. edited and revised manuscript; R.W., X.S., and J.W. approved final version of manuscript.
